# Knowledge, barriers and facilitators of exercise in dialysis patients: a qualitative study of patients, staff and nephrologists

**DOI:** 10.1186/s12882-016-0399-z

**Published:** 2016-11-24

**Authors:** Manisha Jhamb, Mary L. McNulty, Gerald Ingalsbe, Julie W. Childers, Jane Schell, Molly B. Conroy, Daniel E. Forman, Andrea Hergenroeder, Mary Amanda Dew

**Affiliations:** 1 Renal-Electrolyte Division, University of Pittsburgh School of Medicine, 200 Lothrop St, PUH C-1101, Pittsburgh, PA 15213 USA; 2 Department of Psychiatry, University of Pittsburgh, Pittsburgh, PA USA; 3 Community member receiving hemodialysis, Pittsburgh, PA USA; 4 Department of Medicine, Hospice and Palliative Medicine, Division of General Internal Medicine, University of Pittsburgh, Pittsburgh, PA USA; 5 Department of Medicine, Division of General Internal Medicine, University of Pittsburgh, Pittsburgh, PA USA; 6 Geriatric Cardiology Section, Department of Medicine, University of Pittsburgh, Pittsburgh, PA USA; 7 Geriatric Research, Education, Clinical Center, VA Pittsburgh Healthcare System, Pittsburgh, PA USA; 8 Department of Physical Therapy, University of Pittsburgh, Pittsburgh, PA USA; 9 Departments of Psychology, Epidemiology, and Biostatistics, University of Pittsburgh, Pittsburgh, PA USA

**Keywords:** Exercise, Dialysis patients, Qualitative study

## Abstract

**Background:**

Despite growing evidence on benefits of increased physical activity in hemodialysis (HD) patients and safety of intra-dialytic exercise, it is not part of standard clinical care, resulting in a missed opportunity to improve clinical outcomes in these patients. To develop a successful exercise program for HD patients, it is critical to understand patients’, staff and nephrologists’ knowledge, barriers, motivators and preferences for patient exercise.

**Methods:**

In-depth interviews were conducted with a purposive sample of HD patients, staff and nephrologists from 4 dialysis units. The data collection, analysis and interpretation followed Criteria for Reporting Qualitative Research guidelines. Using grounded theory, emergent themes were identified, discussed and organized into major themes and subthemes.

**Results:**

We interviewed 16 in-center HD patients (mean age 60 years, 50% females, 63% blacks), 14 dialysis staff members (6 nurses, 3 technicians, 2 dietitians, 1 social worker, 2 unit administrators) and 6 nephrologists (50% females, 50% in private practice). Although majority of the participants viewed exercise as beneficial for overall health, most patients failed to recognize potential mental health benefits. Most commonly reported barriers to exercise were dialysis-related fatigue, comorbid health conditions and lack of motivation. Specifically for intra-dialytic exercise, participants expressed concern over safety and type of exercise, impact on staff workload and resistance to changing dialysis routine. One of the most important motivators identified was support from friends, family and health care providers. Specific recommendations for an intra-dialytic exercise program included building a culture of exercise in the dialysis unit, and providing an individualized engaging program that incorporates education and incentives for exercising.

**Conclusion:**

Patients, staff and nephrologists perceive a number of barriers to exercise, some of which may be modifiable. Participants desired an individualized intra-dialytic exercise program which incorporates education and motivation, and they provided a number of recommendations that should be considered when implementing such a program.

## Background

Of the 600,000 prevalent end-stage renal disease (ESRD) patients in the US, the vast majority suffer from fatigue, poor sleep, poor physical functioning and frailty [1–4]. These highly debilitating symptoms lead to falls, loss of independence, frequent hospitalizations, premature mortality as well as poor health-related quality of life (HRQOL) [5–9]. Exercise improves aerobic capacity, blood pressure and HRQOL in ESRD patients [10]. Compelling observational data also suggest that regular exercise may improve fatigue, sleep and mortality in hemodialysis (HD) patients [11, 12]. However, <50% of HD patients in the US exercise at least once a week [12]. In fact, the vast majority of these patients often report very low levels of physical activity in general, which is associated with increased symptom burden, poor physical functioning and higher mortality [13, 14].

International data suggest that patients from dialysis facilities that offer exercise programs are 38% more likely to exercise regularly [12]. Unfortunately, in the US, exercise is not part of “standard clinical care” and few facilities offer any kind of exercise program to the patients [12]. This is despite Kidney Disease Outcomes Quality Initiative (KDOQI) guidelines for increasing physical activity in ESRD patients almost a decade ago [15], and over 30 years of research demonstrating safety and benefits of intra-dialytic exercise [16]. Intra-dialytic is ideal as it can be implemented in a setting with rigorous clinical monitoring, does not involve additional time or travel, and may alleviate patient fear of injury [16, 17]. The present challenge involves identification of strategies for translation of research findings into clinical practice [18, 19]. A critical barrier has been the failure to identify an optimal exercise program that meets the needs and preferences of both patients and providers, and is sustainable within the limited resources of the clinical setting [18, 19].

Since dialysis patients, staff and nephrologists are the key stakeholders in the implementation, acceptance and adherence for exercise, especially an intra-dialytic exercise program, it is critical to understand their knowledge, barriers, motivators and preferences. Prior survey-based studies have failed to provide in-depth exploration of participants’ views [20–23]; and the limited qualitative work in this area has mainly focused on specific patient populations or single university-based dialysis units, thus limiting the transferability of results to the majority of dialysis patients and practices in the US [21, 24–26]. We are unaware of any prior qualitative studies that included nephrologists; assessed patients, staff and nephrologists congruently; or sought recommendations from these groups on how to best design an intra-dialytic exercise program.

In this qualitative study, we conducted in-depth interviews with HD patients that were heterogeneous with respect to age, gender and race; dialysis staff at different organizational hierarchy levels; and academic and private nephrologists. Given the recent increased emphasis in the physical activity field on the potential benefits of overall physical activity at any “dose”, as well as emphasis on the benefits of formal exercise training [27], we described exercise as any kind of physical activity such as walking, climbing steps, etc. The primary aim was to elicit participants’ knowledge, barriers and motivators for physical activity in general and specifically for intra-dialytic exercise. The overarching goal was to inform the development of a patient and provider preferred exercise program for HD patients.

## Methods

### Participant recruitment

We recruited ESRD patients ≥18 years on in-center HD 3 times/week for at least 3 months. We used purposive sampling to ensure a sample that reflected a range of important characteristics and ensured adequate number of patients in each of the 4 pre-identified strata – a) sex (men/women); b) age – younger (18–49 years)/older(50–80 years); c) race (White/Black) and d) education level (high school or less/more than high school). Patients were recruited until “saturation”, i.e., no new themes emerged in the interviews. Exclusion criteria included contraindication to exercise (e.g. unstable angina), refractory/untreated psychiatric disease, history of poor adherence to HD treatments, currently in an acute or chronic care facility, life expectancy less than 6 months. After screening the patients through medical chart review, the research team discussed with the dialysis nurse manager and/or social worker at each unit to confirm patient eligibility. Out of the 17 patients initially consented, 1 dropped out due to lack of time availability.

Similar strategies of purposive sampling and thematic saturation were used to recruit dialysis staff at different levels of organization and nephrologists from both academic and private practice setting. Since older nephrologists may be more likely to counsel patients about physical activity [28], we recruited an age-stratified sample [younger (<50 years)/older (≥50 years)]. All participants were recruited from 4 different out-patient Dialysis Clinic, Inc. (DCI) affiliated dialysis units in Western Pennsylvania and identified based on discussion with nurse manager at each dialysis unit. This study followed the Criteria for Reporting Qualitative Research guidelines for conducting and reporting qualitative research [29]. The study was approved by the University of Pittsburgh Institutional Review Board and all subjects provided informed consent.

### Interviews

Members of the research team (MJ, MLM and MAD) developed an interview guide (see Appendix) for semi-structured interviews and this was pretested with a dialysis patient (GI), dialysis unit nurse manager (AHa) and dialysis social worker (GF). The participants were asked to think about exercise as any kind of physical activity such as walking, climbing stairs, etc. MJ, a nephrologist with prior training in qualitative research conceptualized the study. The current literature in this area was extensively reviewed and lack of physical activity/exercise adoption was identified as a critical gap between the research recommendations and usual clinical practice. In order to develop and implement interventions aimed at increasing physical activity in HD patients, this study was conceptualized and designed to gain insights about patient, provider and system level factors involved. GI, a HD patient for 4 years was a key participant in the study design, data interpretation and analysis. All interviews were conducted by MLM (research coordinator) who was trained by MAD, an expert in qualitative analysis. Participants were probed to ensure rich details emerged. The interviews were conducted over the telephone, audiotaped and lasted approximately 20–40 min. They were transcribed verbatim and randomly audited to ensure accuracy.

### Data analysis

Applying grounded theory framework [29], all transcribed interviews were independently coded by MJ and MLM. Initially, open coding was used, in which any and all themes generated were considered in an inductive manner without restricting the focus. The themes and codes that repeatedly emerged were examined to develop focused themes and sub-themes. Inter-coder consensus was established by cross checking, discussing and refining themes until consensus was achieved. In case of disagreement, the differences in coding were resolved through discussion among MJ, MLM and MAD and reviewed by GI. Under each theme, comments from patients, staff and physicians were organized together to evaluate similarities and differences among these groups. For each theme and subtheme, most descriptive representative statements were chosen after discussion among MJ, MLM and MAD and were reviewed by GI.

## Results

We interviewed 16 in-center hemodialysis patients, 14 dialysis staff members and 6 nephrologists (Table 1). The mean (SD) age of patients was 60.1(17) years, 50% were females, 62.5% were blacks and 25% had high school degree or less. This is representative of the HD population in Pittsburgh area (mean age 64 years, 38% females, 41% blacks; data obtained from DCI, 2015). Dialysis staff included 6 nurses, 3 technicians, 2 dietitians, 1 social worker and 2 unit administrators. Nephrologists also represented a diverse group including one third over 50 years of age, 50% female gender and 50% academic nephrologists.

**Table 1:** Participant characteristics

	Patients (n = 16)	Dialysis staff (n = 14)^a^	Nephrologists (n = 6)
Age, yrs [Mean (SD)]	60.1 (17.0)	48.0 (13.2)	47.8 (11.0)
Age ≥50 years, %	12 (75.0%)	7 (50.0%)	2 (33.3%)
Males	8 (50.0%)	1 (7.1%)	3 (50.0%)
Dialysis vintage (yrs) [Median (range)]	3.9 (0.3, 11.1)	-	-
Race			
White	6 (37.5%)	13 (92.9%)	2 (33.3%)
Black	10 (62.5%)	1 (7.1%)	0
Other	0	0	3 (50.0%)
Education		-	-
High school degree or less	4 (25.0%)		
More than high school degree	12 (75.0%)		
Academic practice			3 (50%)

^a^Dialysis staff included 6 nurses, 3 technicians, 2 dietitians, 1 social worker and 2 unit administrators

### Major themes and subthemes

#### Theme 1: Knowledge and perceived benefits of exercise: patients and staff view exercise as beneficial: “It gives me more energy…”

As illustrated in Table 2, majority of the participants’ comments reflected a view that a major benefit of exercise was improved overall perception of health and well-being. Physical health benefits such as improving cardiovascular health, energy level, muscular strength and balance emerged as a prominent sub-theme, especially in the patient interviews. Male patients seemed to focus more on the muscular strength benefits and females on cardio-protective benefits. Interestingly, only a few of the patients commented on the mental health benefits, such as reduced depression and stress, and an increased sense of accomplishment. We found that all but one patient (72 yo White female) and surprisingly one staff member (dialysis technician, 28 yo) reported no knowledge of benefits of exercise. Most patients were unaware or underestimated the recommended frequency and/or duration of exercise:

*“Probably couldn’t be no more than maybe twice a week” [Interview 10, F, 54yo, Black]*

**Table 2:** Knowledge and perceived potential benefits of exercise for dialysis patients

Overall perceived health and well-being	
Patients	• Making you feel better and just making you able to do more things I guess. [Interview 6, F, 64 yo, White] • I wouldn’t be doing quite as well as I am doing now if I wasn’t doing some form of exercise. [Interview 8, M, 65 yo, Black]
Staff	• I just think it might change their whole attitude, like to a more healthier, more compliant attitude, if they start exercising. [Interview 27, 46 yo, Nurse]
Physicians	• Helps release endorphins and makes people feel better overall [Interview 34, M, 42 yo, academic]
Physical Health benefits	
Cardiovascular	
Patients	• Was on three blood pressure pills, now I don’t take any [after exercising regularly] [Interview 4, M, 64 yo, Black]
Staff	• [Exercise during HD would] bring their blood pressure up while they were on the machines, and actually it would hopefully help them lose a little bit more water-weight, if they broke out a little sweat while they were on the machines. [Interview 25, 46 yo, Nurse] • Improved cardiac health, improved cardiac output. For some of our diabetic patients and patients with vascular disease I would think that even some simple exercises would help to improve their vascular health. [Interview 29, 58 yo, Administrator] • mobilization of edema would be another benefit. [Interview 29, 58 yo, Administrator]
Physicians	• One of the biggest morbidities of dialysis is cardio-vascular so improving cardiovascular health……when patients have very low blood pressure [during HD treatment] sometimes it [exercise during HD] helps keep the pressure up [Interview 34, M, 42 yo, academic]
Energy	
Patients	• It gives me more energy. And the more energy I have, the more I feel like doing things. [Interview 12, M, 75 yo, White] • I can go longer than I would be able to go if I wasn’t doing exercise at all. [Interview 8, M, 65 yo, Black]
Staff	• they will have a lot of energy, more energy. That would be a major benefit, [Interview 27, 46 yo, Nurse]
Physicians	• Exercise is very good for helping people to deal with energy level…. [Interview 34, M, 42 yo, academic]
Muscular strength and balance	
Patients	• I don’t have the tightness in my knees [Interview 4, M, 64 yo, Black] • exercise can …….. help with balance…because that’s where your balance comes from [leg and thigh strengthening] [Interview 16, M, 81 yo, Black]
Staff	• I like to keep people moving, the more you move, the more the muscle grows back, the less likely it is for you to have atrophy problems and then its gonna eventually impact your ADLs [activities of daily living]. [Interview 25, 46 yo, Nurse]
Physicians	• Help strength and balance and help them recover if they were ever in a situation where they would fall.[Interview 32, F, 43 yo, private practice] • May allow them to remain independent longer [Interview 31, F, 66 yo, academic]
Miscellaneous	
Patients	• It gives me a better appetite. [Interview 4, M, 64 yo, Black] • I have COPD and exercise can help me breathe better [Interview 16, M, 81 yo, Black]
Staff	• Improving the metabolism …… I think that an improved metabolism could actually help with their nutritional status. [Interview 25, 46 yo, Nurse]
Physicians	• Sometimes it can help relieve muscle cramps because a lot of these patients have cramps [Interview 33, F, 39 yo, private practice] • It helps keep the weight down because a lot of the dialysis patients you know they’re diabetic they could be obese so that helps a lot. [Interview 33, F, 39 yo, private practice] • In some ways I think you would be better off [exercising during HD], because the thing I worry about with exercise is are you releasing potassium into the blood because of the exercises. [Interview 36, M, 39 yo, private practice]
Mental Health benefits	
Patients	• Exercising keeps you on a [reduced] stress level [Interview 10, F, 54 yo, Black]
Staff	• I think it would help as far as depression goes, you know, I think exercise has definitely been shown to lift the mood, elevate your mood.[Interview 17, 67 yo, Nurse] • Maybe a mental health benefit that they’re doing something productive rather than just whiling away time in the dialysis chair. [Interview 29, 58 yo, Administrator] • Maybe improve mental health as they’re accomplishing simple exercise goals, and the satisfaction and accomplishment that comes along with that. [Interview 30, 54 yo, Administrator]
Physicians	• Improved sense of well-being, improved mood, and I think that would be a big quality of life improvement for dialysis patients. [Interview 36, M, 39 yo, private practice] • A lot of these patients they have very sedentary lifestyles and won’t leave home and a large portion of them I would think for them just coming to dialysis is their only you know trip of the outside world, know give them a sense of participating in something [exercise during HD] [Interview 33, F, 39 yo, private practice]

*Abbreviation. yo* years old

#### Theme 2: Reported barriers to exercise: dialysis makes exercise challenging: “When I come home from dialysis, I’m a little bit wiped out”

We identified a number of patient- and system-related or logistic factors that are perceived as barriers (Table 3). Fatigue or lack of energy, especially post-dialysis fatigue was universally cited as the biggest barrier to exercise and was described as “drained”, “just don’t feel like doing anything after HD”. Limitations on lifting weights due to fear of injuring the fistula, inability to do water exercises due to dialysis catheter and time constraints due to dialysis were other major dialysis-related barriers. Several participants commented on the poor overall health and comorbidities such as arthritis, amputations, leg weakness and blindness as common barriers. Fear of falling, lack of counselling by staff and lack of suitable exercise options were some of the other reported barriers.

**Table 3:** Reported Barriers to Exercise

Dialysis-related Fatigue	
Patients	• And usually when I come from dialysis I’m a little bit wiped out and then Yeah, I’d be kind of drained after dialysis [Interview 8, M, 65 yo, Black] • I was very active at one time and now I’m very tired. You can’t make your body do what you want it to do either all the time because you’re tired [Interview 14, F, 68 yo, Black]
Staff	• I know the machine makes them tired, dialysis in general is just you know it’s a tiring disease [Interview 19, 35 yo, Dietitian]
Physicians	• People get really fatigue while on Dialysis and some of them complain of feeling terribly tired. [Interview 32, F, 43 yo, private practice]
Dialysis access	
Patients	• You can’t do weights anymore when you’re on dialysis…[because of fistula] [Interview 5, M, 25 yo, Black] • I have Band-Aids on the sites that were where the needles had been and I don’t want to do anything that would cause them to bust or start bleeding again. So those days I don’t do any exercise. [Interview 8, M, 65 yo, Black] • I was always worried about my fistula if I would fall or something. [Interview 6, F, 64 yo, White] • Based on the fact that I now have a catheter and the fistula, it’s prohibited of me from going back to that [water pool exercise class][Interview 11, F, 65 yo, White]
Non-specific dialysis related factors	
Patients	• I think the dialysis itself takes enough out of them. I know it does for me; when I go home I have such a headache, I lay down and sleep for a couple of hours [Interview 11, F, 65 yo, White]
Time constraints due to dialysis	
Patients	• I usually can only exercise on the days that I don’t go to dialysis, so that really only allows me 2 days a week,[Interview 8, M, 65 yo, Black] • As a result of the dialysis, I have to cram six days of living into three, and so I live alone, so I have to do everything myself [Interview 13, F, 72 yo, White] • Because with my schedule right now I’m trying to get a kidney and I have so many appointments to go to and I have so much going on [Interview 14, F, 68 yo, Black]
Staff	• It’s also about finding what kind of exercises they might like or could fit into their day, just because dialysis already takes up so much time. [Interview 22, 28 yo, Dietitian]
Comorbidities	
Patients	• I have severe arthritis ….. in my knees and in my shoulders [Interview 12, M, 75 yo, White] • Not too many of us are walking without wheelchairs and canes [Interview 15, F, 76 yo, White] • [Other patients] have a lot of other disabilities. One of them has diabetes, he’s blind. Another guy has a double amputation. [Interview 12, M, 75 yo, White] • I’m not sure we have the stamina to continue with exercising for any length of time……, I know what I should be doing but once again what I should be doing and what I can do are 2 different things. There are people of course that are and can only do certain things [Interview 11, F, 65 yo, White]
Staff	• Because of their age and maybe their health too. Some are sicker than others. [Interview 17, 67 yo, Nurse]
Physicians	• A lot of our patients they have Diabetes and they’re old and they can’t see very well and can’t hear very well. They can’t feel their feet very well because they have Neuropathy [Interview 32, F, 43 yo, 1302 academic]
Lack of motivation	
Patients	• There are days you know where you just don’t feel like doing a whole lot so you just don’t do it. Between being tired and having other physical problems too, it’s tough to get somebody motivated enough to want to try to do it. [Interview 12, M, 75 yo, White] • I’d stop exercising and then you know when you stop something you get lazy about it and no motivation [Interview 1, M, 46 yo, Black]
Staff	• The tough part of putting in exercise regularly you know is more of a mental challenge than it is physical [Interview 22, 28 yo, Dietitian] • A lot of our patients are there because they really didn’t take care of their diabetes, hypertension, not very many are interested but some of them are interested in changing their ways and being healthier, but some are just like “Oh well”.[Interview 27, 46 yo, Nurse] • The nature of a chronic illness I think has a set of mental challenges and obstacles all by itself simply because it’s a chronic disease[Interview 30, 54 yo, Administrator]
Physicians	• So yeah there is a fear component and there is a lazy component too. [Interview 32, F, 43 yo, private practice]
Fear of side effects or complications	
Patients	• I have fallen several times because I wasn’t paying attention to what I was doing and I have been lucky so far [Interview 12, M, 75 yo, White]
Physicians	• I have a patient who has Osteoporosis and she’s fallen and broken a hip and now she’s not in to any exercises. …so yeah there is a fear component [Interview 32, F, 43 yo, private practice]
Logistic barriers for exercise	
Patients	• When it’s cold outside, I don’t have as much [exercise/walking][Interview 4, M, 64 yo, Black]
Lack of Staff confidence for counseling	
Staff	• I think I would [be more comfortable with counseling] if I had a guideline to follow or something, I think a little training, in-service training, would be good [Interview 17, 67 yo, Nurse]

*Abbreviation. yo* years old

As with adopting any lifestyle modification, lack of motivation is one of the biggest challenges – and patients, staff and nephrologists readily recognized this. Patients reported that dealing with physical and mental challenges of having a chronic illness and being on dialysis made exercise a low priority for them. Providers felt most patients lacked the motivation to take care of their health and were thus not likely to care about exercising.

#### Theme 3: Reported barriers to intra-dialytic exercise: intra-dialytic exercise should be safe without disrupting usual care: “None of us want to stay there any longer than we have to”

We identified several barriers related to type, safety and feasibility of intra-dialytic exercise (Table 4). As expected, safety concerns regarding blood pressure stability, limitations due to inability to use the access arm, fear of needle dislodgement and infiltration and cramps due to exercise were commonly reported barriers. Only a few patients stated that exercising in front of others was a significant barrier. Several participants talked about limitations related to the ease of use, cost, and storage of exercise equipment. Some of them had prior experience using a stationary pedaling bike during HD and offered reasons for poor adherence - patients felt that the bike was boring, staff felt that it added to their workload. All participants felt that extensive staff involvement in any intra-dialytic exercise program would be impractical. An interesting barrier that emerged was patients’ resistance to changing the routine of dialysis. Although patients described situational reasons for this (e.g., extending time spent at the dialysis center), providers viewed resistance in terms of patients’ personal characteristics, describing some, especially long-term HD patients as “strong-willed”, and introducing any change to dialysis routine as being “an uphill battle”.

**Table 4:** Barriers to intra-dialytic exercise program

Safety-related limitations of Exercise	
Patients	• It might make your blood pressure go up [Interview 2, M, 80 yo, White] • Can’t move that arm, you’re locked into that position for however how long your treatment is. …also on your other free arm you have a blood pressure cuff. So you’re really kind of locked on both sides. Any movement in either one of those arms is gonna cause the machine’s alarm to go off. [Interview 8, M, 65 yo, Black]
Staff	• They could dislodge a needle possibly …. it would also cause them pain, and it could really cause some large damage to their access…the biggest barrier that I could see…. Just moving around too much and cause an infiltrate [Interview 17, 67 yo, Nurse] • If they do too much movement they won’t get a good treatment because they will constantly alarm. [Interview 18, 35 yo, Technician] • Sometimes just moving around causes cramping in a patient [Interview 24, 28 yo, Technician] • When people put their feet down you know blood pressure drops so it would definitely make it a little more challenging as far as making sure that the vitals are staying stabilized. [Interview 24, 28 yo, Technician]
Physicians	• How do you interpret high blood pressures when you’re exercising? I don’t think you can. So it does create some issues that would have to be [addressed] [Interview 36, M, 39 yo, private practice] • People passed out on Dialysis. If they are doing something else that’s directing blood flow elsewhere again it’s just sort of not an ideal combination. If someone’s inclined because they tend to drop their blood pressure, if you’re going to make them exercise you’re going to exacerbate [hypotension] [Interview 32, F, 43 yo, private practice] • They’re almost like sort of tied up to the machine. one hand is immobile so that pretty much you know they just have the use of one hand and we don’t like the patients to be really moving their arm too much because then the needles would move and there is chances of them infiltrating [Interview 33, F, 39 yo, academic]
Limitation related to Type of Exercise and Exercise Equipment	
Patients	• Getting it [stationary bike] up to the chair would be a challenge. It would probably be too big and clunky of a device to put in there [Interview 4, M, 64 yo, Black] • I found that [stationary bike that he used in a past research study] to be very boring, I didn’t like it……….. And I don’t think anybody did because I don’t see anybody doing it now” [Interview 8, M, 65yo, Black]
Staff	• Buying equipment so we would need to get permission with [the administrator] with the budget. I don’t know if we’re going to want to spend all this money on some sort of equipment and have half the patients not use it [Interview 19, 35 yo, Dietitian] • Some kind of equipment becomes cumbersome for patients.[Interview 23, 60 yo, Social worker] • Depending on the exercise, you have space limitations[Interview 28, 58 yo, Nurse] • Some of them used it [stationary bike] for a short time and then they lost interest in it [Interview 29, 58 yo, Administrator]
Physicians	• It would be a little bit expensive because I guess it would involve some sort of equipment. [Interview 31, F, 66 yo, academic]
Lack of privacy during exercising	
Patients	• I don’t like to be what I think is doing performing in front of people because I don’t think I would do it well. [Interview 12, M, 75 yo, White]
Staff	• I think for some people it could be a self-conscious thing and maybe they you know some people don’t like to go to the gym because they don’t want others to see them. [Interview 19, 35 yo, Dietitian]
Impact on staff workload	
Patients	• Yes it would [impact workload] and guarantee they wouldn’t have time to add that to their load. They’re extremely busy the whole time. …so adding one more responsibility to them I don’t think is realistic [Interview 11, F, 65 yo, White]
Staff	• As far as the staff goes I don’t think we would have the time to do any really one-on-one [exercise]. [Interview 18, 35 yo, Technician] • It would have to be very minimal. As our staffing doesn’t provide for helping them exercise. I mean just setting them up for it is you know is extra work. we don’t always have the staff and we don’t always have the time.[Interview 21, 52 yo, Nurse] • With the bicycle[stationary bike]…that did add to our work load and not that it was a bad thing but you know it was a good thing for the patients but….I really don’t have a minute to spare” Interview 28,58 yo, Nurse]
Physicians	• Nurses and techs, they’re on their toes all the time, they are busy and there’s the machines are alarming, someone you know needs attention all the time, and they are not like over-staffed, they are under-staffed so this would be something extra [Interview 33, F, 39 yo, private practice] • There are so many patients per nurse, how attentive can they be when there's another variable in play. [Interview 36, M, 39 yo, private practice]
Resistance to changing dialysis routine	
Patients	• If you did it [exercise in dialysis unit prior to starting HD] none of us want to stay there any longer than we have to….you just don’t want to be there. It’s a mental thing, you just want to get out. [Interview 10, F, 54 yo, Black] • I go [to dialysis] early in the morning so I ain’t gonna be doing no exercise early in the morning I like to sleep during the dialysis mostly [Interview 3, M, 54 yo, Black]
Staff	• The exercising…..we try to encourage them as much as … some people can be very….strong-willed [in adopting changes to their HD routine]. [Interview 18, 35 yo, Technician] • I just don’t see them being willing to, ….. they want to get on the machine, they want to either go to sleep or they want to sit there and do nothing and watch TV.. [Interview 21, 52 yo, Nurse]
Physicians	• It not really part of the culture of during Dialysis….. some of them have been on Dialysis for a long time and they’re used to their routine and they come in and they do whatever they do and ….. I’m not sure that would be completely easy to change…its going to be an uphill battle to get them to exercise right now … they have their routine [Interview 31, F, 66 yo, academic] • A lot of the patients are not receptive to new ideas …..it’s going to be a big change[Interview 33, F, 39 yo, private practice]

*Abbreviation. yo* years old

#### Theme 4: Motivators and facilitators for exercise: motivation for exercise comes from within and from the encouragement of others: “I’m 100% for exercise, I know first-hand that it is beneficial”

A number of patients expressed self-motivation to exercise, arising from either experiencing positive benefits or from recognition of loss of physical fitness after starting dialysis (Table 5). Patients who had accepted dialysis as a lifestyle change seemed to overcome the psychosocial challenges of being more physically active. Participants also identified health incentives and achieving health goals as important motivators to exercise. We also identified a strong trusting relationship of patients with their doctors (primary care and nephrologists) and dialysis staff, and this was a key motivating factor, along with encouragement from family, friends and other dialysis patients. Although most nephrologists offered a number of thoughts on what would motivate patients for an intra-dialytic exercise program (as mentioned below), majority of them did not comment on motivators for overall physical activity.

**Table 5:** Motivators/Facilitators to Exercise

Self-motivation/self-awareness of need to exercise	
Patients	• At one time I wasn’t sure that [exercise] is what I wanted to do, I realized later that’s what I needed to be doing. I’m 100% for exercise, I know first-hand that it is beneficial … anything is better than nothing…. it’s not so much of trying to be the best in the gym or the best in the place just the fact that you’re there. [Interview 8, M, 65 yo, Black] • I hate to use the word but you just suck it up and that’s about it. So the worst thing to do is to just sit around and mope so you don’t do that. ….I feel really guilty when I come home on these cold days and I can’t go out. I feel guilty about that. [Interview 12, M, 75 yo, White] • Every day, every chance and all day we should do something. Even though you’re tired you try to do what you can. You benefit by being able to do a little bit more. It’s a battle and you know you just can’t sit there because that’s not doing you any good [Interview 14, F, 68 yo, Black] • Well I should be doing a lot more, if I could. And I mean mentally, it bothers me that I can’t run up the steps anymore like I used to or it takes me longer to get from the car inside the house. [Interview 4, M, 64 yo, Black]
Staff	• Maybe the end goal of improving their energy, improving how they feel after dialysis, any beneficial factors….like I said energy or stress [Interview 22, 28 yo, Dietitian] • Some of our patients are motivated by health goals, it doesn’t just have to be transplant, if they wrestle with something else health wise and they’re on a path for improvement their own health for some can be motivating as well. [Interview 30, 54 yo, Administrator]
Doctors’ advice	
Patients	• [Doctors advised] that I had to exercise, to lose weight, whatever it was that I could do that would cause me to shed those pounds, that’s what I did [Interview 4, M, 64 yo, Black] • My doctor, the one that I deal with at clinic, I’d do anything he tells me to do. [Interview 12, M, 75 yo, White]
Staff	• The support from the medical practitioners would be critical, I mean these are the same people interacting with them about all aspects of their life and their goals [Interview 30, 54 yo, Administrator]
Staff encouragement	
Patients	• And I have a crew down there at the dialysis clinic that don’t let you do that [sit and mope around]. They encourage it [exercise]. … it’s just a good environment [Interview 12, M, 75 yo, White]
Staff	• I’m a huge proponent [of exercise], if you’re able, I wanna encourage patients to do whatever that I believe and they state is their ability level. [Interview 25, 46 yo, Nurse] • I believe in the use it or lose it mentality. You know whether it’s tough for them to workout sometimes, it’s almost like you have to kinda push through it to reap the benefits… I think there may be …. some form of exercise that almost everyone can do. I definitely think that exercise is important in any way they can fit it in [Interview 22, 28 yo, dietitian]
Physicians	• If the whole team sort of supported them trying it then I think some of them would do that [Interview 31, F, 66 yo, academic]
Support person (family, friend, other dialysis patients)	
Patients	• You gotta have somebody that when you work out, you gotta have somebody to get you motivated, Somebody to push you! [Interview 3, M, 54 yo, Black] • I have friends that I walk with, I have a son that’s 26 and he goes to a gym 3 times a week and he’s always telling me I should exercise [Interview 6, F, 64 yo, White]
Staff	• Yeah that’s usually how our patients are motivated is by lobby discussion [in dialysis unit waiting room] with each other. Yeah usually they’re more receptive to hearing it from another patient [Interview 29, 58 yo, Administrator] • Most have a spouse or a sibling or child or parent that’s working with them in part of their lives. That person’s buy in could be motivating [Interview 30, 54 yo, Administrator]
Type of exercise program	
Patients	• It has to be interesting for you, for you to want to do it. …… If you can find something that you like to do that occupies your mind then you might want to keep it up.[Interview 8, M, 65 yo, Black]
Staff	• Finding what they enjoy you know would be number one [Interview 22, 28 yo, Dietitian]

*Abbreviation. yo* years old

#### Theme 5: Recommendations for intra-dialytic exercise: “It would be good for us mentally to have something else to do there while we are sitting in those chairs”

Participants’ thoughts on why, how and what kind of intra-dialytic exercise program they believed might work are shown in Table 6. A recurring theme was the convenience in terms of saving travel and precious non-dialysis time. Moreover, by adding distraction to the mundane routine of dialysis, participants felt that it would make the time go faster and add value to the dialysis time. They reiterated that since the dialysis unit is a social environment and if a culture of exercise is introduced, it is likely to be well accepted by the patients. However, an individualized engaging program with prior testing of individual’s capabilities was important to the patients. A dialysis unit administrator described his experience with a pilot group physical therapy program in his unit involving an in-center physical therapist:

*“it was well tolerated, it was well accepted … was extremely positive from patients. The anecdotal patients’ feedback was overwhelming, not just one or two, essentially all patients seemed to very much appreciate the attention, the diversion…. the patient response one after another after another totally was “This is fun, I like it, I love the person, I look forward to it”. From our measurements of patient perception, participation, and their feedback, it was a wonderful success.” [Interview 30, 54 yo, Administrator]*

**Table 6:** Recommendations for Intra-dialytic Exercise

WHY intra-dialytic exercise might work? – Provide time distraction and convenience	
Patients	• If you could do it [exercise during dialysis] that would be great. It’s kind of torture sitting in a chair for 3 h. I’ve always questioned why there wasn’t something for us to do there besides sit there. It would be good for us mentally to have something else to do there while we are sitting in those chairs [Interview 10, F, 54 yo, Black]
Staff	• The convenience too, if they were here for 3–4 h, you know, some people get pretty bored…..but if they were able to do some exercise, that would be a way to spend the time. [Interview 17, 67 yo, Nurse] • They would see how much fun other people were having doing it, and if the staff gets into it, just walking around and doing their work, they’ll be playing around with it, and it just makes for a very light and fun afternoon, time will go fast. [Interview 23, 60 yo, Social worker] • You’re not doing it outside of dialysis which is precious time to our patients….. trying something new in a very routine and monotonous process that they live with every week. It may end up that something like that is motivating because it’s different.[Interview 30, 54 yo, Administrator]
Physicians	• There is such a time thing for dialysis itself, it would be nice to somehow turn that time into productivity. I think accessibility would be good… you might have more compliance because you’re stuck there for four hours [Interview 36, M, 39 yo, private practice]
HOW intra-dialytic exercise might work? – Build “Exercise culture” in dialysis unit	
Patients	• I’m the kind of person says that if you can do it, I can do it [if you saw someone else exercise, could that be motivating?] [Interview 12, M, 75 yo, White]
Staff	• It’s that mentality if you see someone else trying to improve themselves and then you kind of like feel bad about yourself …….. And then maybe if they are seeing their friends across the aisle doing it, then that might motivate them to do it as well. [Interview 19, 35 yo, Dietitian] • I would think that it[exercise during dialysis] would hopefully kind of be a chain reaction sort of thing – somebody saw someone doing it, you know, while they were on the machine, and then maybe they can get a couple [of other patients] to follow suit. [Interview 25, 46 yo, Nurse] • If they saw somebody that was on dialysis for many, many years, kind of embracing this new philosophy…..That could possibly light the fire for others. [Interview 25, 46 yo, Nurse] • Most of the staff I think would encourage the patients and have fun with it and help the patient have fun with it. [Interview 26, 41 yo, Nurse]
Physicians	• It’s a social environment and what one person is doing it going to possibly influence another and that can be positive results so other people who see that person exercising may want to do it. If the whole team sort of supported them trying it then I think some of them would do that[Interview 31, F, 66 yo, academic]
WHAT type of intra-dialytic exercise might work? – Individualized, engaging, group activity	
Patients	• I think before the exercise is implemented they should give an example to see how the exercise is, if they would be able to do them and how far they should go because we’re not in that position to do all that they might want us to do [Interview 14, F, 68 yo, Black] • Yeah more flexible type because I can choose the intensities because when I get tired or when I’ve had enough, I’m going to stop. [Interview 4, M, 64 yo, Black]
Staff	• Have a spirited person to try to engage them, and just kind of make it fun. Group activities where you see other people enjoying it and you feel like you want to participate. [Interview 23, 60 yo, Social worker] • Being involved in group activities not necessarily them being pin-pointed you know all by themselves? [Interview 24, 28 yo, Technician]
Physicians	• I can even imagine having different regimens at higher and lower levels where you have the fitter or more stable patients do something that’s more like high level and maybe the less fit do sort of the leg lifts or other exercises. [Interview 36, M, 39 yo, private practice]
WHAT components to include? – Education about exercise	
Patients	• They should put something like that [information on exercise] on the wall [in dialysis unit][Interview 7, F, 43 yo, Black]
Staff	• I think what would be best initially is just to do some pretty heavy education on the benefits. You know especially kind of hitting home the role of exercise with giving them energy… you need to get stronger so you can live on your own again leave the nursing home, get back to home and this is something that can help you reach those goals. [Interview 19, 35 yo, Dietitian]
HOW to motivate? - Incentive to exercise	
Patients	• You have to find some kind of a benefit from what you’re doing or you’re not going to do it and when I do it, I feel good. I feel good about myself. I feel good because I’ve done it. And that’s the only payment you can get from it. [Interview 12, M, 75 yo, White]
Staff	• Maybe testimonials [from patients about benefits of exercise] ….if they could see that [Interview 28, 58 yo, Nurse] • It would be every few months, do some sort of contest or raffle or something they could earn if they do their exercise,[like we did for fluid gains][Interview 19, 35 yo, Dietitian] • I’m sure that [gift cards] would be incentive but that’s costly. Everybody likes money! [Interview 26, 41 yo, Nurse]
Physicians	• Maybe in the beginning offering some sort of encouragement or incentive chance whether it’s you know they can have TV or something …. maybe a raffle or … a prize or something. That’d be key to get them motivated to want to do it. [Interview 34, M, 42 yo, academic]

Despite its initial success, the program failed due to lack of resources to support the in-center exercise staff, thus again emphasizing the need for a less resource intensive program.

Staff and nephrologists suggested offering patients incentives such as prizes or raffles as a way to motivate them to participate in exercise. Interestingly many patients identified positive health benefits and the ability to do more as internal motivators for engaging in exercise. “I feel good because I’ve done it. And that’s the only payment you can get from it [*Interview 12, male, 75 yo*, white].

#### Theme 6: Dialysis staff attitude towards promoting exercise: “I know it can be done and that it can be tolerated”

Since dialysis staff - the nurses, technicians, social workers, dietitians and administrators - are the key front line staff involved in the successful implementation of any intra-dialytic exercise program, we elicited their attitude and willingness to support such a program and the extent of their participation that they thought would be feasible. Majority of staff members believed that intra-dialytic exercise was safe and feasible:

*“There’s clearly a significant population of patients who could participate in some kind of planned exercise during a dialysis treatment, I know that to be possible. I know it can be done and that it can be tolerated” [Interview 30, 54 yo, Administrator]*

Although most of the staff members were supportive of an intra-dialytic exercise program, they strongly felt that their direct responsibility should be limited to patient encouragement, motivation and monitoring. Staff felt that a program that required minimal staff assistance could be easily accommodated and would not add to their workload.

*“I don’t see it [encouraging exercising] being a huge issue …. monthly education with them on that [exercise] like I do with the diet and you know adding a little bit of work but I don’t think it would cumbersome.” [Interview 19, 35 yo, dietitian]*

*“I don’t know if that would, you know, interfere with us making sure that they’re okay ‘cause we do every half hour check-ups basically we’re making our rounds to every patient, making sure that they’re okay, checking all of their vitals.…….. I wouldn’t see it [exercise during dialysis] being that much of a hassle.” [Interview 24, 28 yo, technician]*

## Discussion

In this qualitative study, we identified the knowledge, barriers and motivators to exercise in dialysis patients by interviewing HD patients, dialysis staff and nephrologists. Although the need for promoting exercise participation in dialysis patients is widely recognized, as is evident by the National Kidney Foundation guidelines [15], the lack of an exercise routine in a majority of dialysis patients uncovers the critical need for understanding exercise-related barriers and preferences. Our study takes a novel approach of addressing these important questions by including a heterogeneous sample of all the key stakeholders – patients, dialysis staff and nephrologists. Additionally, by eliciting their recommendations for an intra-dialytic exercise program, it lays the foundation for developing a patient and provider preferred program that is more likely to have long term acceptability and adherence.

Most dialysis patients viewed exercise as beneficial, consistent with prior studies [20]. However we identified a critical gap in patients’ knowledge of mental health benefits of exercise, thus providing a target for intervention. We found that patients unanimously underestimated the exercise goals, which per the KDOQI guidelines should be at least 30 min of moderate-intensity exercise on most, and preferably all days of the week [15]. This extends the findings of Painter et al. who found that even dialysis staff were largely unaware of these guidelines [26]. Although these guidelines imply the notion that quantification of exercise and reaching a critical threshold may be important as a key metric of efficacy, the recent focus in the physical fitness field has shifted to greater emphasis on overall physical activity in lieu of the burdensome sentiment that only 150 min/week can or will determine benefit [30, 31]. It is especially important to impart this knowledge to dialysis patients and providers as many of our patients may be unable or unwilling to engage in higher doses or duration of exercise. More importantly, the emphasis should be on increasing overall physical activity rather than participating in formal exercise training program. Most HD patients may be unable or unwilling to participate in formal exercise training, but may be more inclined and able to modify their day to day routine so as to incorporate more physical activity and reduce their sedentary behavior. However, such a shift in focus not only needs revision of national guidelines, but also educational efforts to increase awareness among the health care providers so that they can appropriately counsel and motivate their patients.

Confirming findings from previous studies, a major barrier identified by patients and providers was fatigue [5, 20, 25]. At the same time, improvement in energy level was recognized as a benefit of exercise by all participants. Lack of energy due to the disease or the dialysis treatment itself may lead to low physical activity, which in turn may perpetuate fatigue and the vicious cycle of inactivity continues. Since the majority of dialysis patients value improvement in fatigue, even more than improvement in survival [32], more research is needed to find how and what kind of exercise may lessen the experience and impact of fatigue in these patients.

Although some prior studies have examined barriers to exercise [20, 24–26], an additional important focus of our study was identifying factors and behaviors that would motivate patients to participate and adhere to exercise. Self-awareness of benefits of exercise was a key element, similar to what Kontos et al. reported in a predominantly white Canadian cohort [25]. Accepting dialysis as part of life, starting slow, doing something rather than nothing, desire to achieve pre-dialysis physical health, experiencing positive benefits from exercise and achieving health goals were other motivators cited by the patients. Incorporating communication skills such as motivational interviewing can be useful to elicit these patient motivators to reinforce and encourage adherence to an exercise program, as has been shown in dialysis and non-dialysis patients [10, 33]. However, how to incorporate such interventions in a clinical setting with minimal additional resources or costs remains a challenge.

As with any life-changing disease, patients relied heavily on their health-care providers and social network for support, encouragement and guidance for exercise. More importantly, it was evident that there is an interactive contextual influence of the provider and clinic setting on the patients’ attitudes towards exercise. Patients who perceived the staff to be supportive, were more likely to have a positive attitude towards exercise *"…I have a crew down there at the dialysis clinic that don't let you do that [sit around and mope]. They encourage it [exercise]…it's just a good environment."* (Table 5)*.* Given that prior studies have shown that assessment of physical function is not part of routine practice [26], nor are dialysis staff or nephrologists confident in counseling patients about exercise [22, 28], a major change in provider training and practice patterns is called for. Additionally, increased involvement of health professionals with specialized training in exercise prescription such as physical therapists may be needed.

Because so much of dialysis patients’ lives revolve around the process of getting dialysis and non-dialysis time is precious, we wanted to explore what participants thought about doing exercise during dialysis. Almost all participants felt that an intra-dialytic exercise program would be preferred to exercising at other times, as well as feasible. Important barriers related to safety, limitations of type of exercise and impact on staff workload emerged. An intriguing set of barriers pertained to patients’ apparent resistance to changing dialysis routine. However, both patients and professionals felt that building a culture of exercise and obtaining commitment from long-time dialysis patients may inspire others to follow. Novel approaches for building such social support systems within and across dialysis units by integrating technology, such as competitions and rewards based on number of steps using wearable accelerometers, should be explored in future studies.

An important consideration for long-term adherence was participants’ desire for a multi-modal intra-dialytic exercise training program that incorporated exercise, educational and motivational components. It was viewed as very important to have an engaging exercise program which would incorporate elements that could be individualized to the patient’s health status and ability. In general, dialysis staff had a supportive attitude towards intra-dialytic exercise, but it would be crucial to minimize their active responsibility and workload in such a program. Dietitians and technicians seemed most willing to advocate for such a program.

A limitation of our study may be that although we continued to conduct interviews until we reached what we saw as thematic saturation, we may have failed to capture additional thoughts and beliefs that might have emerged from more interviews. However, we used purposive sampling to select a heterogeneous group of participants with key characteristics in each group which is likely to be reflective of a broader population and thus would facilitate the capture of a wide range of relevant views, concerns and beliefs. Moreover, we incorporated feedback from a dialysis patient, unit nurse manager and social worker in developing the interview guide, thus including a broad-range of questions and probes. However, although the patients were representative of the Pittsburgh HD population, they were older, included more blacks and did not represent diverse ethnicity as compared to the United States HD population (mean age 54 years, 43% females, 31% blacks, 17% Hispanics) [34]; which may limit generalizability of our findings. Also, all participants were recruited from a single large dialysis organization (LDO, DCI) and may not represent the practices at other LDOs. The findings from our study may not be applicable to the home dialysis patients. However, in the US, more than 10 times as many ESRD patients receive HD treatments at a clinic as those who do peritoneal dialysis and home HD combined [34]. Thus, our findings are likely to be relevant to more than 90% of the ESRD patients in the US. Lastly, we did not include representatives of large dialysis organizations (LDOs) in the US in our study. We believe that the onus rests on the nephrology research and clinical community to devise innovative ways of increasing physical activity and delivering intra-dialytic exercise to HD patients, with minimal additional costs and resources. However, not involving LDOs as stakeholders may be a limitation of this study and is very important for future research.

## Conclusion

Given the paucity of interventions to improve the HRQOL and physical well-being of patients on chronic HD and the promise of exercise therapy, there is a critical need to optimize exercise participation in these patients. Our study identifies important barriers to exercise, several of which may be modifiable, as well as factors that may motivate HD patients to exercise. Patients and providers favor a multi-modal program that includes individually-adaptable intra-dialytic exercises, education and motivation Understanding and incorporating the preferences and devising novel ways to incorporate these in a cost- and resource- effective manner is the key to successful implementation of such a program. Future research is needed to design such a program and evaluate its impact on patient outcomes.

## Appendix

### Interview guide

This contains a draft of questions and probes that were used for qualitative interviews. These questions were used only as a guide to aid in starting the conversation.

Interview Script:

Exercise or any kind of physical activity can improve physical fitness and health of patients on dialysis. But it is hard for dialysis patients to do regular exercise. We want to find out what are your thoughts about exercise. By exercise, we mean any kind of physical activity, including walking, climbing stairs, etc. We are interested in finding out barriers and motivators for exercise in dialysis patients. This information will help us in designing an exercise program that would address these barriers and incorporate patient and provider preferences. We will ask you some questions. Please tell us as much detail as you can.

Draft of planned questions and probes for qualitative interviews

{NOTE: These questions are as a guide to aid in starting the conversation. Based on participants’ responses, further exploration of their thoughts and opinions is encouraged}

Questions for patients:

1. What do you see as the benefits of doing exercise in general?
2. How much exercise per day or per week should you be doing to protect your health and keep healthy?
3. Would you prefer doing exercise during your dialysis session or at home on your non-dialysis day? Why?
4. If we have an exercise program in your dialysis unit, would you be interested in doing exercise during your dialysis session? Why or why not?
5. Have you ever participated in any exercise program during dialysis session? If yes, how what exercise did you do and how was your experience?
6. Will exercising during dialysis in front of other people bother you? Why or why not?
7. Do you think exercise during dialysis has any side effects? Why?
8. Do you think you would be scared or anxious to do exercise while you are on dialysis? Or would it make you less anxious knowing you are in a supervised environment with health care professionals around? Why?
9. Are you worried about any injury due to exercise? Why? Any past experiences?
10. Are you worried about how exercise during dialysis will affect staff workload? In what way?
11. If we show you exercise videos during dialysis that you can follow, would that be something you would be interested in doing?
12. Would you like exercise sessions with a fixed set of exercises or a more flexible program in you can choose type and intensity of exercise? Why?
13. What kind of leader or instruction giver would you prefer to see in the exercise videos?
14. What phrases during exercise will keep you motivated to finish a session of exercise?
15. When doing exercise during dialysis, you will not be able to move the arm which has the fistula or catheter. Would you like to exercise that arm prior to dialysis or at home? Why?
16. Would you like to get more information on benefits and safety of exercise for dialysis patients, in general?
17. Would you be interested in watching videos that give you more information on benefits and safety of exercise? If yes, would you prefer to watch these videos at home or during dialysis?
18. It may be hard for patients to have motivation to get started or continue on an exercise program. Would you be open to meeting a motivational counselor who can help you resolve any concerns that you might have and motivate you to exercise? If not, why? What other things would motivate you?
19. Would you be interested in watching video clips about other people with illnesses who exercise? Would this motivate you to exercise? If yes, would you prefer to watch these videos at home or during dialysis?

Questions for dialysis staff:

1. Do you know the benefits of exercise for HD patients? Can you tell us some of the benefits
2. Do you see exercise during dialysis as feasible for patients? Why or why not?
3. What concerns do you have about risks of exercise during dialysis?
4. What do you think will motivate patients to do exercise doing dialysis?
5. If patient exercise during dialysis, do you think it will add to your work? What would be acceptable as “additional work”?
6. Do you feel comfortable counselling patients to exercise? Have you had any training in this? If not, what would make you more comfortable?
7. What barriers do you see for implementing an intra-dialytic exercise program?

Questions for nephrologists:

1. Do you know the benefits of exercise for HD patients? Can you tell us some of the benefits
2. Do you see exercise during dialysis as feasible for patients? Why or why not?
3. What concerns do you have about risks of exercise during dialysis?
4. What do you think will motivate patients to do exercise doing dialysis?
5. Do you feel comfortable counselling patients to exercise? Have you had any training in this?
6. What would make you more comfortable in counselling patients to exercise?
7. What barriers do you see for implementing an intra-dialytic exercise program?

## Abbreviations

DCI: Dialysis clinic, Inc.
ESRD: End-stage renal disease
HD: Hemodialysis
HRQOL: Health-related quality of life
KDOQI: Kidney disease outcomes quality initiative
LDO: Large dialysis organization
SD: Standard deviation
